# Revisiting judging reliability in taekwondo freestyle Poomsae: implications for AI-supported evaluation

**DOI:** 10.3389/fpsyg.2026.1881296

**Published:** 2026-09-07

**Authors:** Min-Woo Jeon, Hong-Suk Kim, Ji-Yong Park, Hye-Soo Cho

**Affiliations:** 1 Department of Taekwondo, College of Physical Education, Kyung Hee University, Yongin-si, Republic of Korea; 2 Department of Sports Science, Hanyang University ERICA, Ansan-si, Republic of Korea

**Keywords:** fairness, freestyle Poomsae, inter-rater agreement, judging, test-retest reliability

## Abstract

**Background:**

Before artificial intelligence-based judging systems can be applied to Taekwondo Poomsae, it is necessary to understand which performance components human judges can identify consistently and which remain difficult to evaluate reliably. This study examined reliability in freestyle Poomsae judging to identify evaluation components with stable and unstable human scoring.

**Methods:**

Ten internationally certified Taekwondo Poomsae referees evaluated ten official competition videos twice, separated by a one-week washout period. Systematic session effects were evaluated with separate linear mixed-effects models estimated by restricted maximum likelihood using the SPSS MIXED procedure. Session was specified as a fixed effect, judge and video as crossed random intercepts, and the two observations within each judge-video pair as repeated measurements with an unstructured residual covariance matrix. Fixed-effect inference used Satterthwaite denominator degrees of freedom. Test–retest stability was evaluated for identical judge-video pairs, with primary emphasis on the absolute-agreement ICC because systematic session effects were detected. Inter-rater agreement was evaluated separately by session using two-way random effects, single rating absolute-agreement and consistency ICCs with 95% confidence intervals and Kendall’s W. Item-specific analyses were exploratory, and no multiplicity adjustment was applied.

**Results:**

The total score increased by 0.304 points, 95% CI [0.187, 0.421], t (99) = 5.177, *p* < 0.001. At the nominal 0.05 level, exploratory item-specific increases were observed for jumping side kick (*β* = 0.057, *p* < 0.001), acrobatic kicking technique (*β* = 0.021, *p* = 0.003), and expression of energy (*β* = 0.047, *p* = 0.003); the increase for basic movements and practicability did not reach the 0.05 threshold (*p* = 0.053). Absolute-agreement test–retest ICCs ranged from 0.039 to 0.848 and were excellent for harmony and the total score. All single-rating inter-rater ICC(A,1) point estimates were below 0.40. For the total score, ICC(A,1) was −0.007, 95% CI [−0.018, 0.036], in the first session and 0.060, 95% CI [0.010, 0.224], in the second session.

**Conclusion:**

Systematic session effects, absolute test–retest agreement, relative consistency over time, and inter-rater agreement represent distinct aspects of judging reliability. Evaluation components with poor single-judge agreement require clearer operational definitions and further validation before computational or AI-assisted scoring is considered.

## 1 Introduction

In aesthetic sports such as gymnastics, figure skating, and dancesport, where athletes’ movements and artistic expression are evaluated, judging largely relies on the subjective assessments of experienced judges. Bias within judging systems in aesthetic sports has been consistently examined through analyses of historical competition data (Bučar et al., 2012; Leskošek et al., 2010; Lockwood et al., 2005; Bučar Pajek et al., 2014; McGee, 2019; Sato, 2025). For instance, athlete reputation and popularity (Findlay and Ste-Marie, 2004), memory-influenced biases (Ste-Marie et al., 2001), differences in judges’ visual search and error-detection expertise (Flessas et al., 2015), and physical conditions such as judges’ viewing angles (Dallas et al., 2011) have been identified as key factors affecting scoring reliability.

To address subjectivity and enhance the reliability of judging, several sports have implemented structural improvements in their scoring systems. For example, artistic gymnastics has significantly increased the reliability of judges’ scores by strictly separating difficulty and execution and by introducing detailed scoring guidelines that specify kinematic criteria for all technical elements (Atiković et al., 2011; Pajek et al., 2013). Similarly, figure skating introduced the International Judging System (IJS) following the judging scandal at the 2002 Winter Olympics, separating technical elements from program components to reduce judges’ discretion and enhance objectivity (Looney, 2012). These efforts to objectify evaluation systems in traditional aesthetic sports are particularly important because previous studies have consistently reported higher reliability for technical scores than for artistic scores (Lockwood et al., 2005; Bučar Pajek et al., 2014).

However, patterns of scoring reliability have been reported to vary depending on the clarity of the evaluation criteria and the characteristics of each sport. In particular, sports such as dancesport and hip-hop dance, which lack clearly defined kinematic evaluation criteria, continue to exhibit lower reliability than gymnastics (Premelč et al., 2019; Sato, 2022). Notably, Sato’s (2025) analysis of breaking, which was included as an official sport in the Paris 2024 Olympic Games, revealed not only lower judging reliability than that reported for gymnastics but also a counterintuitive finding: the artistic domain (e.g., creativity), which is generally expected to exhibit greater variability and lower reliability, demonstrated higher reliability than the technical domain. A common implication across these studies is that in the absence of clear operational definitions (i.e., explicit guidelines), the reliability of technical evaluations can substantially decline despite their presumed objectivity.

Accordingly, the present study moves beyond conventional findings from traditional aesthetic sports, where technical scores tend to demonstrate higher reliability than artistic scores. Instead, it focuses on two issues: the contrasting findings observed in emerging expressive sports such as breaking, where the absence of clear kinematic evaluation criteria has been associated with lower reliability in the technical domain, and the limitations underlying these findings (Sato, 2025). A comprehensive review of prior studies suggests that judging reliability in aesthetic sports varies depending on the use of absolute or relative evaluation systems, the characteristics of the sport, and the availability of detailed scoring guidelines.

Therefore, rather than relying solely on the observed statistical results, recommendations from previous studies concerning the development of independent and clearly defined rating scales that reflect the distinct characteristics of the artistic and technical domains (Bučar Pajek et al., 2014), the systematic control of cognitive and physical sources of bias that undermine judging reliability (Dallas et al., 2011; Flessas et al., 2015; Sato, 2025), and calls for methodological rigour in emerging Olympic disciplines (Yang and Whatman, 2025) should be considered.

The development and limitations of judging systems in aesthetic sports have important implications for Taekwondo freestyle Poomsae, which is currently being considered for inclusion as an official event in the 2028 Los Angeles Olympic Games. In the past, Taekwondo sparring (Kyorugi) relied entirely on judges’ visual assessments for scoring, resulting in frequent controversies over officiating decisions. However, technologies such as the Protector and Scoring System (PSS), which uses sensors to measure impact force and accuracy, and AI-based video review systems (Zhang et al., 2025), have replaced purely visual impact judgments with sensor-based measurement and technology-assisted review (Choi et al., 2021). These technological advancements in Kyorugi provide a successful example of improved judging reliability in Taekwondo. From this perspective, the recent emergence of freestyle Poomsae as a key Taekwondo discipline highlights the need for quantitative research to promote fairness, a fundamental value of the Olympic Games, and guide improvements in its judging system.

Accordingly, this study aimed to evaluate the overall test–retest stability and interrater agreement of the total score and to explore item-specific reliability patterns within the technical skills and presentation domains of Taekwondo freestyle Poomsae judging. Systematic session effects were additionally examined to determine whether repeated ratings were affected by changes in mean score levels over time. Through this approach, the study seeks to identify structural limitations of the judging system that have previously been discussed only at a conceptual level.

Furthermore, the study aims to propose institutional improvements to enhance the objectivity and fairness of the Taekwondo freestyle Poomsae judging system and provide practical implications for judge training and the development of evaluation guidelines.

To achieve these objectives, the following research questions were established:

*RQ1:* What levels of test–retest stability and interrater agreement are observed for the total score in Taekwondo freestyle Poomsae judging?

*RQ2:* What item-specific patterns of test–retest stability and interrater agreement are observed among the subcomponents within the technical skills domain?

*RQ3:* What item-specific patterns of test–retest stability and interrater agreement are observed among the subcomponents within the presentation domain?

## 2 Materials and methods

### 2.1 Participants

To address the limitation that prior studies of judging reliability in dancesport and breaking have primarily focused on inter-rater reliability based on single-time-point data (Premelč et al., 2019; Sato, 2025), this study employed a test–retest design with a washout period to reduce potential memory and carryover effects when identical Taekwondo freestyle Poomsae performances were evaluated repeatedly.

Given the practical constraints in recruiting highly qualified raters, previous studies examining judging reliability in aesthetic sports have typically relied on expert panels of approximately ten judges (Atiković et al., 2011; Premelč et al., 2019; Sato, 2025).

Based on these considerations, the present study selected ten expert judges holding international referee certifications, considering the repeated-measures design and the sample sizes used in previous studies. The study protocol was approved by the Institutional Review Board of Hanyang University (HYUIRB-202605-006), and all procedures were conducted in accordance with the Declaration of Helsinki.

The video stimuli consisted of publicly available official competition footage organized by the Korea Taekwondo Association. The Institutional Review Board approval included the use of identifiable performance footage as experimental stimuli. Because the study involved secondary analysis of existing competition footage without direct interaction with the athletes or collection of additional personal information, the requirement for individual informed consent was waived by the Institutional Review Board. No identifiable images from the video materials are reproduced in this manuscript.

### 2.2 Experimental design

In this study, ten video recordings of athletes who achieved first place in officially recognized competitions organized by the Korea Taekwondo Association, including national team selection trials, were edited and used as experimental stimuli. First-place performances were intentionally selected to ensure that all judges evaluated routines of comparable competitive quality performed under official competition conditions. This design allowed the reliability of judging to be examined under standardized high-level performance conditions while minimizing variability attributable to large differences in competitive performance. Each video represented a different athlete, and only one performance from each athlete was included in the study.

The experiment was conducted over a two-week period with two repeated measurements. To control for potential confounding variables affecting test–retest reliability, such as practice and recency effects, a one-week washout period was implemented between the two evaluation sessions. Within each evaluation session, all judges viewed the video clips in the same presentation order to maintain identical viewing conditions across judges. However, to minimize potential carryover and memory effects, the presentation order of the ten video clips was changed between the first and second evaluation sessions.

During the evaluation, international judges used the 2024 freestyle Poomsae scoring criteria based on the World Taekwondo (WT) Poomsae Competition Rules (World Taekwondo, 2024). The scoring system consisted of two domains: technical skills (6.0 points) and presentation (4.0 points), for a total of 10 points. The technical skills domain was further divided into the level of difficulty of kicking techniques and basic movements and practicability. The level of difficulty included five components: jumping side kick (height), multiple kicks in a jump (number of kicks), spinning kicks (degree of rotation), consecutive kicks, and acrobatic techniques. The presentation domain consisted of four components: creativity, harmony, expression of energy, and music and choreography. Each subcomponent was scored on a scale ranging from 0.0 to 1.0 in increments of 0.1; the detailed scoring criteria are presented in Table 1.

**Table 1:** Allotted scoring criteria and operational definitions for freestyle Poomsae.

Scoring criteria	Details of scoring criteria	Point	Operational definition
Technical skills (6.0)	Level of difficulty of foot techniques	5.0	Points are awarded for the overall difficulty and performance level of foot techniques across five evaluation areas.
	Jumping side kick		Points are awarded based on the performance level of the jumping side kick and the height of the jump.
	Multiple kicks in a jump		Points are awarded based on the performance level of multiple kicks and the number of kicks executed during a single jump.
	Gradient of spins in a spin kick		Points are awarded based on the performance level of the spin kick(s) and the degree of rotation, such as 180°, 360°, 540°, or 720°.
	Kyorugi style consecutive kicks		Points are awarded based on the mastery and performance level of consecutive Kyorugi-style kicks in a row; the number of consecutive kicks is limited from seven (7) to ten (10).
	Acrobatic kicking technique		Points are awarded based on the mastery and performance level of kicking techniques performed with acrobatic movements.
	Basic movements & practicability	1.0	Points are awarded for accuracy in Taekwondo basic movements and for the completeness of the connection between attacks and defenses, as well as the overall practicability of the freestyle Poomsae performance.
Presentation (4.0)	Creativity	4.0	Points are awarded based on the originality of actions and the creative composition of the Poomsae performance.
	Harmony		Points are awarded based on the harmony among performance components, such as music, choreography, and attire; in pair and team events, harmony among performers is also evaluated.
	Expression of energy		Points are awarded in accordance with the evaluation standard for expression of energy used in recognized Poomsae.
	Music & choreography		Points are awarded based on how well the music and choreography fit together in the overall performance.
Maximum points		10.0	The highest possible total score is 10.0 points.

### 2.3 Data analysis

#### 2.3.1 Systematic session effects

For each evaluation item and the total score, a separate linear mixed-effects model was fitted to the data in long format. Because 10 judges rated 10 videos in two sessions, each model included 200 observations from 100 unique combinations of judge and video. Analyses were conducted using the MIXED procedure in IBM SPSS Statistics for Windows version 31.0. Session was entered as a fixed effect and coded as 0 for the first evaluation and 1 for the second evaluation. Crossed random intercepts for judge and video were specified in separate RANDOM subcommands using a variance components covariance structure. Because each judge rated every video in both sessions, the two observations associated with each combination of judge and video were treated as repeated measurements. Specifically, session was specified in the REPEATED subcommand, each unique combination of judge and video was specified as the subject and an unstructured residual covariance matrix was applied [COVTYPE(UN)]. Models were estimated using restricted maximum likelihood, whereas the fixed session contrast represented the estimated change from the first evaluation to the second evaluation. This contrast was reported as β together with its standard error, 95% confidence interval, t statistic, degrees of freedom approximated using the Satterthwaite method and two-sided *p* value. The total score model represented the overall analysis, whereas the item-specific models were exploratory. Therefore, no adjustment for multiple testing was applied and the item-level *p* values were interpreted as nominal.

Test–retest stability was evaluated using the scores from the first and second sessions for each identical combination of judge and video, resulting in 100 paired observations for each item. ICCs were estimated using the RELIABILITY procedure in SPSS under a two-way mixed-effects formulation. Combinations of judge and video were treated as random targets, whereas the two sessions were treated as fixed measurement occasions. ICC(A,1) measured absolute agreement for a single rating and ICC(C,1) measured consistency for a single rating, whereas ICC(C,2) measured the consistency of the mean of the two session-specific ratings. All coefficients were reported with 95% confidence intervals. Because the mixed-effects analyses identified systematic differences between sessions, ICC(A,1) was designated as the primary index of test–retest stability because it is sensitive to changes in score level. ICC(C,1) and ICC(C,2) were retained as supplementary indices of relative consistency. Importantly, ICC(C,2) refers only to the average of the two repeated session ratings and does not represent an average-measure interrater coefficient.

Interrater agreement was estimated separately for the first and second evaluation sessions. Within each session and for each scoring item, the 10 videos were treated as targets and the 10 judges were treated as raters. Because the inferential target was the agreement of a score assigned by an individual judge drawn from the relevant population of judges, a two-way random-effects model in the single-rating form was used. Using the RELIABILITY procedure in SPSS, we calculated the absolute-agreement coefficient for a single rating [ICC(A,1), numerically equivalent to ICC(2,1)] and the consistency coefficient for a single rating [ICC(C,1)]. Each coefficient was reported with a 95% confidence interval based on the F distribution. Interrater coefficients based on average ratings [ICC(A,10) and ICC(C,10)] were not reported because they estimate the reliability of the mean score assigned by the complete panel of 10 judges rather than the agreement of a score assigned by an individual judge, which was the estimand of the present study. Specifically, ICC(A,1) assessed whether judges assigned the same numerical score to a video, whereas ICC(C,1) assessed whether judges preserved the relative ordering of the videos. Kendall’s coefficient of concordance (W) was also calculated as a rank-based measure of concordance, and its significance was evaluated using the chi-square approximation.

ICC values below 0.40 were classified as poor, values from 0.40 to 0.74 as acceptable and values of 0.75 or higher as excellent. Similarly, Kendall’s W values below 0.40 were classified as low, values from 0.40 to 0.49 as moderate, values from 0.50 to 0.70 as good and values above 0.70 as excellent. All statistical tests were two-sided with α = 0.05. Finally, the item-level analyses were exploratory and no adjustment for multiple testing was applied.

## 3 Results

### 3.1 Systematic session effects between the first and second evaluations

The estimated session coefficients were positive for all scoring items, indicating a general tendency toward higher scores in the second evaluation. The total score increased by 0.304 points (SE = 0.059, 95% CI [0.187, 0.421], *t* (99) = 5.177, *p* < 0.001). At the nominal 0.05 level, the exploratory item-specific analyses indicated increases for jumping side kick (*β* = 0.057, SE = 0.011, 95% CI [0.036, 0.078], *t* (99) = 5.395, *p* < 0.001), acrobatic kicking technique (*β* = 0.021, SE = 0.007, 95% CI [0.007, 0.035], *t* (99) = 2.998, *p* = 0.003), and expression of energy (*β* = 0.047, SE = 0.016, 95% CI [0.016, 0.078], *t* (99) = 3.026, *p* = 0.003). The estimated increase for basic movements and practicability did not reach the nominal 0.05 threshold (*β* = 0.054, SE = 0.028, 95% CI [−0.001, 0.109], *t* (99) = 1.958, *p* = 0.053) (Table 2).

**Table 2:** Systematic session effects estimated using linear mixed-effects models.

Domain	Evaluation item	1st M +/− SD	2nd M +/− SD	Mean change	β (session)	SE	95% CI	t (df)	*p*
Technical skills	Jumping side kick	0.620 +/− 0.142	0.677 +/− 0.132	0.057	0.057	0.011	[0.036, 0.078]	5.395 (99)	< 0.001
Technical skills	Multiple kicks in a jump	0.605 +/− 0.221	0.627 +/− 0.242	0.022	0.022	0.027	[−0.031, 0.075]	0.821 (99)	0.414
Technical skills	Gradient of spins in a spin kick	0.733 +/− 0.092	0.745 +/− 0.093	0.012	0.012	0.009	[−0.007, 0.031]	1.269 (99)	0.208
Technical skills	Kyorugi-style consecutive kicks	0.678 +/− 0.054	0.686 +/− 0.067	0.008	0.008	0.007	[−0.006, 0.022]	1.157 (99)	0.250
Technical skills	Acrobatic kicking technique	0.716 +/− 0.080	0.737 +/− 0.092	0.021	0.021	0.007	[0.007, 0.035]	2.998 (99)	0.003
Technical skills	Basic movements & practicability	0.572 +/− 0.237	0.626 +/− 0.223	0.054	0.054	0.028	[−0.001, 0.109]	1.958 (99)	0.053
Presentation	creativity	0.657 +/− 0.182	0.692 +/− 0.123	0.035	0.035	0.022	[−0.008, 0.078]	1.626 (99)	0.107
Presentation	Harmony	0.686 +/− 0.129	0.695 +/− 0.140	0.009	0.009	0.007	[−0.006, 0.024]	1.216 (99)	0.227
Presentation	Expression of energy	0.631 +/− 0.202	0.678 +/− 0.158	0.047	0.047	0.016	[0.016, 0.078]	3.026 (99)	0.003
Presentation	Music & choreography	0.607 +/− 0.226	0.646 +/− 0.227	0.039	0.039	0.027	[−0.015, 0.093]	1.440 (99)	0.153
Overall	Total score	6.505 +/− 0.966	6.809 +/− 0.941	0.304	0.304	0.059	[0.187, 0.421]	5.177 (99)	< 0.001

For each outcome, *N* = 200 observations from 100 judge-video pairs. Separate linear mixed-effects models included session as a fixed effect, crossed judge and video random intercepts specified with a variance-components structure, and repeated session measurements within judge-video pairs modeled with an unstructured residual covariance matrix. Models were estimated by restricted maximum likelihood. Fixed-effect inference used Satterthwaite denominator degrees of freedom. β is the estimated change from the first to the second evaluation. Item-specific *p* values are nominal because these analyses were exploratory and no multiplicity adjustment was applied.

No nominally significant session effects were observed for multiple kicks in a jump, gradient of spins in a spin kick, Kyorugi-style consecutive kicks, basic movements and practicability, creativity, harmony, or music and choreography. Because no multiplicity adjustment was applied, the item-specific *p* values should be interpreted as exploratory rather than as confirmatory evidence. The findings therefore indicate a systematic increase in the total score and nominal item-level evidence for selected components; they should not be interpreted as demonstrating an absence of session effects for the remaining components.

### 3.2 Test–retest stability of identical judge-video ratings

Table 3 reports test–retest stability for identical judge-video pairs across the two sessions. Because systematic session effects were detected, interpretation focused primarily on absolute agreement. Absolute stability was excellent for harmony [ICC(A,1) = 0.848, 95% CI [0.782, 0.895] and the total score ICC(A,1) = 0.773, 95% CI [0.593, 0.865]]. Jumping side kick [ICC(A,1) = 0.650], gradient of spins in a spin kick [ICC(A,1) = 0.474], acrobatic kicking technique [ICC(A,1) = 0.652], and expression of energy [ICC(A,1) = 0.614] showed acceptable absolute stability.

**Table 3:** Test–retest stability of identical judge-video ratings.

Domain	Evaluation item	ICC(A,1) [95% CI]	ICC(C,1) [95% CI]	ICC(C,2) [95% CI]	Interpretation
Technical skills	Jumping side kick	0.650 [0.418, 0.784]	0.704 [0.590, 0.791]	0.826 [0.742, 0.883]	Acceptable
Technical skills	Multiple kicks in a jump	0.333 [0.147, 0.496]	0.332 [0.146, 0.495]	0.498 [0.254, 0.662]	Low
Technical skills	Gradient of spins in a spin kick	0.474 [0.308, 0.612]	0.476 [0.309, 0.614]	0.645 [0.472, 0.761]	Acceptable
Technical skills	Kyorugi-style consecutive kicks	0.352 [0.169, 0.512]	0.353 [0.169, 0.513]	0.522 [0.289, 0.678]	Low
Technical skills	Acrobatic kicking technique	0.652 [0.512, 0.755]	0.669 [0.545, 0.764]	0.802 [0.705, 0.867]	Acceptable
Technical skills	Basic movements & practicability	0.277 [0.090, 0.446]	0.283 [0.092, 0.453]	0.441 [0.169, 0.624]	Low
Presentation	creativity	0.039 [−0.154, 0.231]	0.040 [−0.157, 0.234]	0.077 [−0.372, 0.379]	Low
Presentation	Harmony	0.848 [0.782, 0.895]	0.848 [0.782, 0.895]	0.918 [0.878, 0.945]	Excellent
Presentation	Expression of energy	0.614 [0.466, 0.727]	0.633 [0.499, 0.737]	0.775 [0.666, 0.849]	Acceptable
Presentation	Music & choreography	0.283 [0.095, 0.452]	0.285 [0.095, 0.455]	0.444 [0.173, 0.626]	Low
Overall	Total score	0.773 [0.593, 0.865]	0.810 [0.731, 0.868]	0.895 [0.844, 0.930]	Excellent

The target unit was the identical judge-video pair (*n* = 100 pairs per item), and the two sessions were treated as fixed measurement occasions in a two-way mixed-effects formulation. ICC(A,1) = single-rating absolute agreement; ICC(C,1) = single-rating consistency; ICC(C,2) = consistency of the mean of the two session-specific ratings. Because systematic session effects were detected, the interpretation column is based primarily on ICC(A,1). Values are presented with 95% confidence intervals.

In contrast, multiple kicks in a jump [ICC(A,1) = 0.333], Kyorugi-style consecutive kicks [ICC(A,1) = 0.352], basic movements and practicability [ICC(A,1) = 0.277], creativity [ICC(A,1) = 0.039], and music and choreography [ICC(A,1) = 0.283] showed poor absolute test–retest stability. Consistency estimates were similar or moderately higher; for the total score, ICC(C,1) was 0.810 and ICC(C,2) was 0.895. The difference between the total-score absolute-agreement and consistency coefficients indicates that the systematic shift in score level reduced absolute reproducibility even though the relative ordering of judge-video ratings was largely preserved.

Inter-rater agreement was estimated separately for each evaluation session (Table 4). All single-rating ICC(A,1) and ICC(C,1) point estimates were below 0.40 in both sessions, indicating poor agreement for a score assigned by an individual judge. In the first evaluation, ICC(A,1) estimates ranged from −0.049 to 0.202 and ICC(C,1) estimates ranged from −0.065 to 0.243. For the total score, absolute agreement was essentially null [ICC(A,1) = −0.007, 95% CI [−0.018, 0.036]], as was consistency [ICC(C,1) = −0.031, 95% CI [−0.074, 0.123]].

**Table 4:** Inter-rater agreement calculated separately for each evaluation session.

Domain	Evaluation item	1st ICC(A,1) [95% CI]	1st ICC(C,1) [95% CI]	1st Kendall W (p)	2nd ICC(A,1) [95% CI]	2nd ICC(C,1) [95% CI]	2nd Kendall W (p)
Technical skills	Jumping side kick	−0.020 [−0.038, 0.046]	−0.046 [−0.081, 0.084]	0.083 (0.593)	−0.026 [−0.060, 0.096]	−0.033 [−0.076, 0.116]	0.171 (0.080)
Technical skills	Multiple kicks in a jump	0.000 [−0.037, 0.134]	0.000 [−0.059, 0.196]	0.156 (0.122)	−0.033 [−0.056, 0.053]	−0.051 [−0.084, 0.070]	0.190 (0.047)
Technical skills	Gradient of spins in a spin kick	0.051 [−0.027, 0.287]	0.057 [−0.031, 0.309]	0.153 (0.129)	0.016 [−0.027, 0.168]	0.027 [−0.046, 0.252]	0.118 (0.304)
Technical skills	Kyorugi-style consecutive kicks	0.111 [0.005, 0.392]	0.122 [0.005, 0.418]	0.231 (0.014)	0.077 [−0.012, 0.332]	0.088 [−0.014, 0.363]	0.225 (0.016)
Technical skills	Acrobatic kicking technique	0.119 [0.021, 0.385]	0.169 [0.032, 0.484]	0.264 (0.005)	0.139 [0.035, 0.408]	0.238 [0.075, 0.567]	0.268 (0.004)
Technical skills	Basic movements & practicability	−0.012 [−0.031, 0.062]	−0.029 [−0.073, 0.127]	0.153 (0.131)	0.112 [0.018, 0.370]	0.166 [0.030, 0.479]	0.300 (0.001)
Presentation	creativity	0.030 [−0.031, 0.230]	0.038 [−0.040, 0.274]	0.182 (0.060)	0.240 [0.087, 0.559]	0.299 [0.116, 0.630]	0.299 (0.001)
Presentation	Harmony	0.202 [0.062, 0.514]	0.243 [0.079, 0.573]	0.244 (0.009)	0.195 [0.060, 0.502]	0.253 [0.085, 0.583]	0.287 (0.002)
Presentation	Expression of energy	−0.006 [−0.047, 0.139]	−0.008 [−0.063, 0.177]	0.116 (0.316)	0.019 [−0.034, 0.197]	0.026 [−0.047, 0.249]	0.152 (0.135)
Presentation	Music & choreography	−0.049 [−0.071, 0.033]	−0.065 [−0.090, 0.033]	0.041 (0.930)	0.002 [−0.041, 0.153]	0.003 [−0.058, 0.201]	0.152 (0.133)
Overall	Total score	−0.007 [−0.018, 0.036]	−0.031 [−0.074, 0.123]	0.152 (0.133)	0.060 [0.010, 0.224]	0.225 [0.067, 0.553]	0.350 (< 0.001)

Within each session, the 10 videos were treated as targets and the 10 judges as raters in a two-way random-effects model. ICC(A,1), equivalent to ICC(2,1), is the single-rating absolute-agreement coefficient; ICC(C,1) is the single-rating consistency coefficient. F-distribution-based 95% confidence intervals are reported. Average-rating coefficients [ICC(A,10) and ICC(C,10)] are not shown because the reliability of the 10-judge panel mean was not the study estimand. Kendall’s W quantifies rank-based concordance. Negative ICC estimates indicate agreement no better than expected from the estimated variance components. Because only 10 video targets were available, confidence-interval width should be considered when comparing items or domains.

In the second evaluation, ICC(A,1) estimates ranged from −0.033 to 0.240 and ICC(C,1) estimates ranged from −0.051 to 0.299. Creativity had the largest single-rating point estimates [ICC(A,1) = 0.240, 95% CI [0.087, 0.559]; ICC(C,1) = 0.299, 95% CI [0.116, 0.630]], followed by harmony ICC(A,1) = 0.195, 95% CI [0.060, 0.502]; ICC(C,1) = 0.253, 95% CI [0.085, 0.583]. Nevertheless, all point estimates remained below the prespecified threshold for acceptable agreement. For the total score, absolute agreement remained poor ICC(A,1) = 0.060, 95% CI [0.010, 0.224], although consistency was somewhat higher ICC(C,1) = 0.225, 95% CI [0.067, 0.553].

Kendall’s W values were low for all individual scoring items. For the total score, Kendall’s W increased from 0.152 (*p* = 0.133) in the first evaluation to 0.350 (*p* < 0.001) in the second evaluation. This indicates greater rank-based concordance in the second session, but the magnitude remained low. Because only 10 video targets contributed to each inter-rater ICC, several confidence intervals were wide and spanned values from negligible to moderate agreement. Accordingly, apparent differences among the item-level estimates should not be interpreted as definitive differences in interrater agreement among the scoring components.

## 4 Discussion

The primary aim of this study was to distinguish the components of freestyle Poomsae evaluation whose reliability may be improved through refined operational definitions from those that may require broader changes in judging procedures. These findings may also provide preliminary evidence for considering where AI-supported tools could potentially assist human judges in future research. Bias and subjectivity within judging systems in aesthetic sports have long been central topics of research (Bučar Pajek et al., 2014; Bučar et al., 2012; Leskošek et al., 2010; Lockwood et al., 2005). However, prior studies on judging in major aesthetic sports such as dancesport, breaking, and artistic gymnastics have primarily focused on inter-rater agreement based on single-time-point competition data (Atiković et al., 2011; Premelč et al., 2019; Sato, 2025). To address this limitation, the present study extended the methodological scope of previous research by employing a repeated-measures design in which international judges evaluated identical Taekwondo freestyle Poomsae performances with a one-week washout period. This approach enabled not only the examination of inter-rater differences but also the empirical assessment of intra-rater stability over time (Ste-Marie et al., 2001). The revised statistical structure further distinguished systematic session effects, test–retest stability, and inter-rater agreement as separate analytical questions.

At the level of the total score, the same judge reproduced their evaluations with excellent stability across the one-week interval despite a systematic upward shift in the second session. In contrast, agreement between individual judges remained poor in both sessions and showed only a modest improvement in rank concordance at the second evaluation. This systematic increase indicates that certain components may be susceptible to leniency drift, recalibration, familiarity, or memory-related effects. Comparable phenomena have been documented in other aesthetic sports. For example, judges’ prior processing of a routine can bias their subsequent evaluations of the same performance (Ste-Marie et al., 2001), and serial-position effects have been reported in juried competitions (Bruine de Bruin, 2005). Moreover, repeated exposure to a stimulus reliably increases evaluative liking (Zajonc, 1968; Bornstein, 1989), which may partly account for the generally higher scores in the second session.

Importantly, the findings of the present study suggest that instability in freestyle Poomsae judging is not merely attributable to judges’ lack of expertise or experience but is fundamentally linked to differences in the clarity and structural definition of evaluation criteria. This interpretation is consistent with recent findings reported in Olympic breaking (Sato, 2025).

Traditional research in aesthetic sports has generally assumed that technical scores demonstrate higher reliability than artistic scores due to the presence of objective indicators (Bučar Pajek et al., 2014; Lockwood et al., 2005). For instance, artistic gymnastics achieves high judging reliability by providing detailed scoring codes that clearly define difficulty and deduction criteria for all performance elements (Atiković et al., 2011; Bučar et al., 2012; Pajek et al., 2013). The item-level heterogeneity observed in the present study is consistent with recent research on Olympic breaking, which showed that even ostensibly technical criteria may demonstrate limited reliability when they are insufficiently operationalized (Sato, 2025).

Within the technical skills domain, item-specific patterns emerged. The same judge reproduced the jumping side kick, the gradient of spins in a spin kick, and the acrobatic kicking technique with acceptable stability, whereas multiple kicks in a jump, Kyorugi-style consecutive kicks, and basic movements and practicability were reproduced poorly. In addition, no technical subcomponent reached acceptable agreement between judges. Although the World Taekwondo (WT) rules require objective indicators such as the number of kicks performed in jumping techniques, they do not provide specific kinematic anchors—such as knee extension angle or target height—that define what constitutes a valid kick (World Taekwondo, 2024). The findings of the present study suggest that the absence of sufficiently operationalized criteria may reduce judging consistency. In particular, the poor reproducibility of components that require rapid enumeration of near-simultaneous actions such as multiple kicks in a jump suggests that such counting tasks may exceed the temporal resolution of unaided real-time observation. Artistic gymnastics has addressed this demand by delegating enumeration to codified difficulty tables and video review (Bučar et al., 2012; Leskošek et al., 2010). This interpretation is supported by previous studies reporting that even highly experienced judges rely on individual perceptual judgments when objective criteria are insufficiently defined (Heinen et al., 2012; Sato, 2022; Yee and Hoong, 2013). The findings of the present study further suggest that judging is not solely a matter of subjective evaluation but is significantly influenced by how perceptual–motor information, such as joint angles and flight time, is structured and presented within the rules. This view is consistent with social-cognitive accounts of sports officiating, in which scores are understood as products of constructive perception, memory, and inference rather than as direct readouts of performance (Plessner and Haar, 2006; Plessner et al., 2023). In other words, the clearer and more explicitly defined these observable performance indicators are, the greater the consistency among judges and, consequently, the higher the reliability of scoring.

A similarly item-specific pattern characterized the presentation domain. Harmony and expression of energy were reproduced stably by the same judge, whereas creativity and music and choreography were not, and between-judge agreement was limited for all presentation components. Harmony showed excellent test–retest stability, suggesting that some global impression-based judgments may be applied consistently over time (Heinen et al., 2012; Plessner and Haar, 2006). In particular, the low reliability of music and choreography indicates that judges may not share a sufficiently common operational standard for evaluating the fit between music and movement composition (Premelč et al., 2019; Sato, 2022). Unlike harmony, which may be judged as an overall impression of performance, this component requires judges to simultaneously evaluate multiple artistic elements, such as musical synchronization, movement composition, and the integration of music with movement. Because these elements involve greater subjective interpretation and are not operationally defined in sufficient detail in the current rules, judges may apply different interpretations to the same performance, which may partly explain the relatively low reliability observed for this component. It is also worth noting that the artistic domain showed higher reliability than the technical domain in Olympic breaking, which employs a comparative judging format (Sato, 2025), whereas creativity showed poor temporal stability under the absolute scoring format of freestyle Poomsae. This contrast suggests that the evaluation format itself as well as the clarity of criteria may condition the reliability of artistic judgments.

Beyond the refinement of scoring criteria, these findings carry practical implications for judge management and for the prospective role of AI. First, periodic judge calibration using anchor performances with consensually agreed scores may help mitigate the session-related score drift observed in this study. Longitudinal monitoring of individual judges’ scoring behavior, which has been institutionalized in artistic gymnastics (Heiniger and Mercier, 2021), could serve the same purpose. The findings also indicate where AI-based support could begin. In this study judges were least consistent at reproducing the count of multiple kicks in a jump over time and at agreeing with one another on components such as the jumping side kick. These are precisely the quantities that automated systems already measure in neighboring sports. For example, an AI-based judging support system has operated in artistic gymnastics since 2019 (Fujitsu, 2023), sensor-based scoring is used in Kyorugi and AI-assisted video review has shown feasibility (Choi et al., 2021; Zhang et al., 2025), execution scores in diving, vaulting, and figure skating can be estimated from competition video (Pirsiavash et al., 2014; Parmar and Morris, 2019; Xu et al., 2020), and Taekwondo unit techniques can be recognized automatically (Lee and Jung, 2020). In contrast, creativity and music and choreography, which showed the lowest temporal stability and limited between-judge agreement in this study, resist operational definition, and their observed unreliability suggests that individual human ratings do not yet provide a stable criterion for training or validating an algorithm. Accordingly, any introduction of AI should follow a stepwise order in which automated measurement of operationally definable components is applied first, assistive functions such as video review and automated counting are considered next, and extension to the definition-resistant presentation components is deferred until consensus-based criteria have been validated. Throughout this process, holistic judgments such as harmony, which judges in this study reproduced most consistently, should remain with human judges.

## 5 Conclusion

In conclusion, for Taekwondo freestyle Poomsae to achieve recognized fairness on the international stage, including the Olympic Games, the operational definitions of each evaluation component should be refined with caution. Based on the findings of the technical domain, deduction-based criteria specifying observable performance conditions, such as joint angles, flight time, and landing stability, may be considered as one possible approach to improving scoring consistency. However, such criteria should not be regarded as the only solution, and excessive specification could risk reducing the artistic and expressive qualities that characterize freestyle Poomsae. Therefore, future rule refinement should balance measurable technical indicators with the artistic essence of the event. The development of AI-supported judging systems should proceed only after human judging reliability, operational definitions, and machine-detectable movement features have been examined together.

Furthermore, future research should extend to meta-analyses encompassing judging reliability across related martial arts disciplines, such as karate kata (Clark, 2022) and recognized Taekwondo Poomsae. Building on the present findings, future research on AI-assisted judging should first validate automated measurements of kinematically definable components such as jump height, kick count, and rotation angle against the judgments of expert panels. It should then construct consensus-based reference standards from aggregated and repeated expert ratings for the components on which single judges proved unreliable. Only on that basis should researchers examine whether computational indicators of properties such as the synchronization between music and movement (Li et al., 2021) can reproduce expert consensus in the presentation domain. Such a research program would provide the empirical foundation required to transform subjective and impression-based judging into a more scientific and data-driven evaluation system.

## 6 Limitations

This study has certain limitations. As the analysis was conducted in a controlled video-based evaluation environment, it does not fully capture the complex contextual factors present in actual competitions. Previous studies have shown that judging in aesthetic sports can be influenced not only by performance criteria but also by factors such as judges’ viewing angles and positions (Dallas et al., 2011), athlete reputation (Findlay and Ste-Marie, 2004), and physical appearance, consistent with broader evidence that attractiveness cues systematically shape person perception (Cunningham et al., 1990; Pawlowski et al., 2000; Tovée et al., 1999). As ambiguity in evaluation criteria increases, judges may rely more on such external schemas and holistic impressions (Plessner and Haar, 2006; Sato, 2025). Therefore, future studies should examine the interaction effects of physical competition environments on judging reliability.

An additional limitation of this study is that only first-place performances were included. Although this design reduced variability associated with differences in competitive level, it may also have restricted the range of performance quality and compressed score variance. Because ICC estimates are influenced by between-target variance, the observed reliability coefficients may not fully represent judging reliability across the complete spectrum of freestyle Poomsae performances. Future studies should therefore include performances from a broader range of competitive levels, including high-, mid-, and lower-ranked routines, to examine judging reliability across the full scoring range.

## Data Availability

The raw data supporting the conclusions of this article will be made available by the authors, without undue reservation.
